# Percentile-based slope-constrained linear interpolation for robust imputation of highly volatile PM2.5 time series

**DOI:** 10.1016/j.mex.2026.103859

**Published:** 2026-03-13

**Authors:** Sawet Somnugpong, Narut Butploy, Kanokwan Khiewwan

**Affiliations:** Computer Technology Program, Faculty of Industrial Technology, Kamphaeng Phet Rajabhat University, Kamphaeng Phet 62000, Thailand

**Keywords:** Slope-Constrained, PM2.5 Time Series, Missing Data Imputation, Robustness Analysis, Air Quality Monitoring Systems, Percentile-Based Constraint

## Abstract

Reliable reconstruction of missing observations is essential for environmental time-series analysis, particularly for highly volatile air-quality indicators such as PM2.5. Although linear interpolation is widely used for short-gap imputation due to its simplicity and computational efficiency, it does not explicitly regulate slope dynamics and may produce physically implausible transitions in rapidly fluctuating data. This study proposes a percentile-based slope-constrained linear interpolation method that estimates a slope threshold from the empirical distribution of historical first-order differences and applies a sequential constraint during interpolation to prevent unrealistic gradient changes. The approach requires only a single data-driven parameter and maintains linear computational complexity.

• Data-driven slope threshold estimated from the percentile distribution of historical first-order differences.
• Sequential slope constraint applied to prevent unrealistic gradient transitions during interpolation.
• Linear-time method that preserves the simplicity of standard interpolation while improving reconstruction accuracy.

## Graphical abstract

**Figure:**
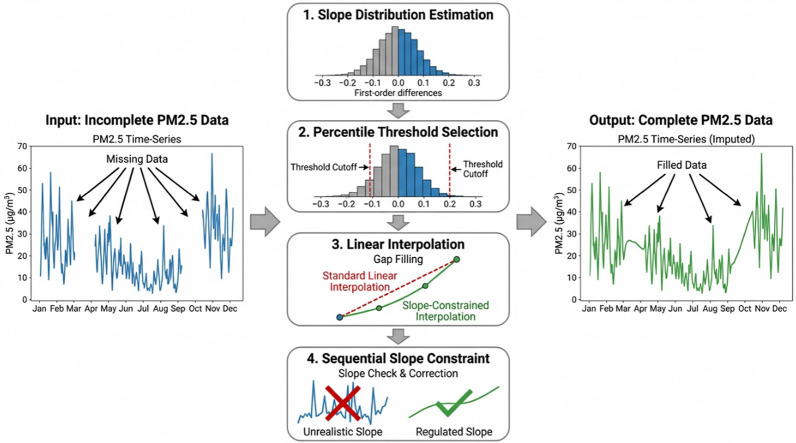

## Specifications table

**Table:** 

**Subject area**	Computer Science
**More specific subject area**	Time Series Analysis, Missing Data Imputation and Robustness Analysis
**Name of your method**	Percentile-Based Slope-Constrained Linear Interpolation
**Name and reference of original method**	Linear interpolation / spline interpolation methods [1,2]
**Resource availability**	PM2.5 Dataset from Pollution Control Department Ministry of Natural Resources and Environment Thailand https://pcd.gdcatalog.go.th/dataset/

## Background

Air pollution has become a major environmental concern worldwide, particularly fine particulate matter (PM2.5), which poses significant risks to human health. Exposure to PM2.5 has been linked to respiratory diseases, cardiovascular conditions, and increased premature mortality rates. Consequently, reliable air quality data are essential for environmental monitoring, public health assessments, and policy development [3]. However, obtaining complete and consistent PM2.5 datasets remains challenging because monitoring stations frequently experience missing observations due to sensor malfunction, equipment maintenance, power limitations, or communication failures. These missing data issues can compromise data reliability and introduce significant errors in long-term trend analysis and exposure assessment [4]. Therefore, accurate missing data imputation is an important preprocessing step before further environmental analysis can be conducted.

Linear interpolation and linear spline interpolation are among the most widely used techniques for handling missing values in environmental time series. These methods are popular because of their simplicity, numerical stability, and computational efficiency, and they are often recommended for short-gap imputation in air quality datasets [5,6]. Several studies have confirmed that linear-based methods remain robust and stable compared with more complex techniques when dealing with short-term missing segments [7,8]. However, linear interpolation does not explicitly regulate the slope behavior of reconstructed time series. When PM2.5 concentrations change rapidly, this limitation may lead to physically implausible transitions between observed data points.

To address this issue, some studies have explored higher-degree polynomial splines, such as quadratic or cubic interpolation, to improve smoothness and derivative continuity. Nevertheless, numerical interpolation research indicates that higher-degree polynomials are prone to overshooting and oscillation, particularly when data are missing at random or when the underlying time series exhibits strong volatility [[9], [10], [11], [12]]. Increasing model complexity does not always guarantee better imputation performance and may even reduce stability in highly dynamic environmental datasets.

Alternative approaches based on statistical and dynamical models, including state-space models, spectral methods, and Kalman filtering, have also been applied to air quality data reconstruction [[13], [14], [15]]. While these approaches can effectively model uncertainty, they often require complex model specification and parameter tuning. More recently, machine learning and deep learning methods such as Long Short-Term Memory (LSTM) networks have been introduced for PM2.5 imputation [16]. Although these models can achieve strong predictive performance, they typically require large training datasets, higher computational cost, and offer limited interpretability, which may limit their applicability in operational environmental monitoring systems [14,17].

However, existing interpolation-based PM2.5 imputation studies rarely incorporate an explicit statistical constraint on the slope distribution of reconstructed values. To address this gap, this study introduces a percentile-based slope constraint integrated with linear interpolation, enabling controlled gradient reconstruction while maintaining the computational simplicity required for operational air-quality monitoring systems.

## Method details

### Data preparation

The dataset used in this study was obtained from the Pollution Control Department (PCD) of Thailand, serving as a statistical repository sourced from automated air quality monitoring stations in the Bangkok Metropolitan Area. The data is structured as a univariate time series from a single point-source monitoring station. Typically, such datasets exhibit non-linear behavior, seasonal fluctuations, and are influenced by both meteorological factors and anthropogenic activities [3,4,9]. To ensure the reliability of the baseline values, the data underwent a quality control process aligned with the guidelines of the World Meteorological Organization (WMO) [18]. The dataset spans a full calendar year [19], comprising 366 daily average PM2.5 readings. To quantify the degree of volatility within the dataset, the Coefficient of Variation (CV) was employed. The CV serves as a standardized measure of relative variability, allowing for a consistent comparison of fluctuations across different datasets [20,21]. The interpretation and calculation of this variance are based on equation (1).

(1) $$CV=\frac{\sqrt{\frac{1}{N-1}\sum_{i=1}^{N}{\left(x_{i}-\mu \right)}^{2}}}{\frac{1}{N}\sum_{i=1}^{N}x_{i}}$$

Interpretation of CV is categorized into three levels: low variability, CV<0.2 moderate variability 0.2<CV<0.5 and high variability: CV > 0.5. The measurement of variance in this study yielded a Coefficient of Variation (CV) of 1.0595. This can be interpreted as the time-series data used in this experiment having high volatility.

### Missing data generation

**Fig. 1:**
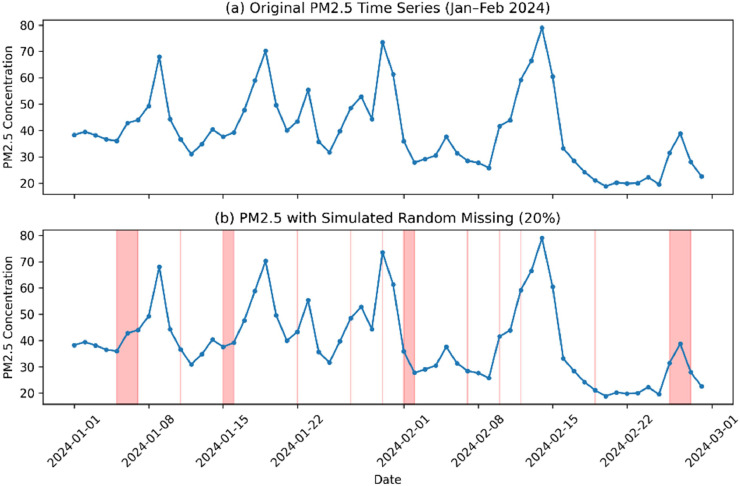
Example of the dataset used in the experiment. (a) Original PM2.5 time series covering a three-month period. (b) Example of simulated missing values with a 20% missing rate, highlighted by red-shaded regions.

This study employs the Missing Completely at Random (MCAR) simulation to introduce gaps into the original dataset. The simulation is conducted to evaluate the robustness of the imputation methods under different levels of data loss. This simulation approach is a widely used standard in air quality imputation research [7,8,22]. The selection of the MCAR mechanism allows for the assessment of method performance without interference from structural biases related to the specific locations of the missing data (Fig. 1).

To test the robustness of the proposed method, data missing rates were generated using a progressive scale. The minimum missing rate was set at 5% of the total data and increased in increments of 5% up to a maximum level of 50%. This progressive missing approach is designed to reflect real-world scenarios, ranging from highly stable monitoring systems to cases where unexpected issues cause severe data damage. This approach of scaling missing rates is recommended by several comparative studies [[23], [24], [25]]. Furthermore, the random selection of missing data positions was repeated multiple times, and the average of the evaluation metrics was used to ensure stability and reliability of the results.

### Estimation and imputation procedure under percentile-based slope-constraint

3.1) The slope distribution is estimated using the first-order differences of consecutive observed values in the PM2.5 time series. The first-order difference is defined as:

(2) $$\Delta x_{i}=x_{i}-x_{i-1}$$

The absolute slope magnitude is then calculated as:

(3) $$s_{i}=\left|\Delta x_{i}\right|$$

For a dataset containing N observed values, this process produces N−1 slope estimates. These slopes are used to construct the empirical slope distribution of the time series.

Next, the value τ is calculated as a threshold to determine whether the interpolation slope at any given point is anomalous. To obtain this threshold, we apply the percentile-based threshold principle, which has been reported as an effective method for distinguishing normal behavior from extreme events [10,22,26].

(4) $$\tau =Percentile_{p}\left(\left\{s_{i}\right\}\right)$$

A sensitivity analysis was conducted to examine percentile levels ranging from 80% to 99%. The results indicate that the 95^th^ percentile provides the best balance between preserving natural variability and preventing unrealistic gradient changes. In this dataset, the 95^th^ percentile corresponds to a slope magnitude of τ = 15.70 µg/m³ per day.

3.2) The next step involves initiating data interpolation using the standard linear interpolation method [1,2], which provides a numerically stable and computationally efficient baseline for reconstructing short missing segments in environmental time series. In this study, the classical linear interpolation framework is extended by introducing a percentile-based slope constraint to regulate unrealistic gradient transitions generated during the interpolation process. The process is applying equation (5) to every missing data point located between the two observed points under consideration, $\left(t_{a},x_{a}\right)$ and $\left(t_{b},x_{b}\right)$.

(5) $${\hat{x}}_{k}^{\left(lin\right)}=x_{a}+\frac{t_{k}-t_{a}}{t_{b}-t_{a}}\left(x_{b}-x_{a}\right)$$

3.3) Subsequently, the slope of the points generated through linear interpolation is calculated using equation (6)

(6) $$\Delta {\hat{x}}_{k}={\hat{x}}_{k}^{\left(lin\right)}-{\hat{x}}_{k-1}$$

The condition for applying the slope-constraint at any given point is as follows: if $|\Delta {\hat{x}}_{k}|>\tau$, the slope will be clipped using equation (7). Otherwise, the value will be defined as ${\hat{x}}_{k}={\hat{x}}_{k}^{\left(lin\right)}$

(7) $${\hat{x}}_{k}={\hat{x}}_{k-1}+sign\left(\Delta {\hat{x}}_{k}\right).\tau$$

The $sign(\Delta {\hat{x}}_{k})$ is utilized to determine the direction of the slope. The proposed method aims to constrain the slope magnitude by increasing or decreasing it while maintaining its original direction. Therefore, whether to increase or decrease the value is determined by the condition in equation (8). Following this, equation (7) is applied to calculate the exact magnitude of the slope adjustment for each interpolated point.

(8) $$sign\left(\Delta {\hat{x}}_{k}\right)=\{\begin{array}{c} \Delta {\hat{x}}_{k}>0\to \;\\ \Delta {\hat{x}}_{k}=0\to \;\\ \Delta {\hat{x}}_{k}<0\to \; \end{array}\begin{array}{c} +1\\ 0\\ -1 \end{array}$$

The operational procedure can be summarized as shown in Algorithm 1

**Algorithm 1:** Slope - constrained linear interpolation.

**Input:** 1. Time series ${\left\{\left(t_{i},x_{i}\right)\right\}}_{i=1}^{N}$ where $x_{i}$ may contain missing values 2. Percentile threshold p
**Output:** Reconstructed time series ${\hat{x}}_{i}$
**Step 1: Compute Empirical Slope Threshold** 1.1 Compute first-order differences from observed data: $\Delta x_{i}=x_{i}-x_{i-1}$ 1.2 Compute absolute slopes: $s_{i}=\left|\Delta x_{i}\right|$ 1.3 Estimate percentile threshold: $\tau =Percentile_{p}\left(\left\{s_{i}\right\}\right)$
**Step 2: *Perform Linear Interpolation*** For each missing segment between observed points $\left(t_{a},x_{a}\right)$ and $\left(t_{b},x_{b}\right)$ 2.1 For each missing time $t_{k}$between $t_{a}$and $t_{b}$: ${\hat{x}}_{k}^{\left(lin\right)}=x_{a}+\frac{t_{k}-t_{a}}{t_{b}-t_{a}}\left(x_{b}-x_{a}\right)$
**Step 3: Apply Slope Constraint (Clipping)** For each reconstructed point ${\hat{x}}_{k}^{\left(lin\right)}$: *3.1 Compute interpolated slope:* $\Delta {\hat{x}}_{k}={\hat{x}}_{k}^{\left(lin\right)}-{\hat{x}}_{k-1}$ if $|\Delta {\hat{x}}_{k}|>\tau$ then ${\hat{x}}_{k}={\hat{x}}_{k-1}+sign\left(\Delta {\hat{x}}_{k}\right).\tau$ else ${\hat{x}}_{k}={\hat{x}}_{k}^{\left(lin\right)}$
**Step 4: Return Reconstructed Series** Return the final reconstructed time series ${\hat{x}}_{i}$ after applying both the interpolation and slope constraint procedures.
Overall complexity: O(N)

From Algorithm 1, the overall process can be described as follows: when a data gap occurs, an initial estimation is calculated using standard linear interpolation. Subsequently, the system enters a validation phase to determine if the resulting interpolated slope exhibits any structural implausibility. This is performed by analyzing the slope at each specific point; if the slope exceeds the predefined boundaries, an adjustment process is triggered. This process recalibrates the imputed values to align with either the upper or lower slope limits while strictly preserving the original direction of the slope.

**Fig. 2:**
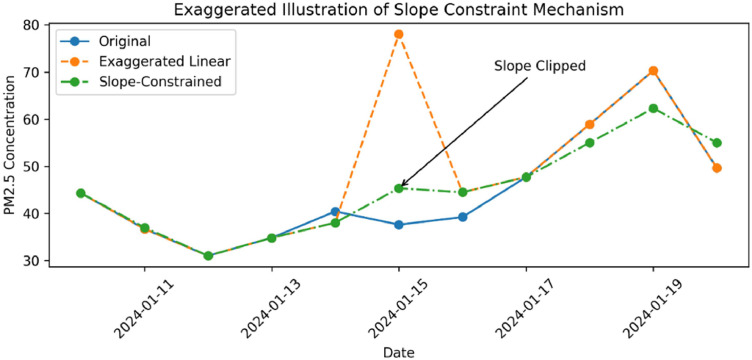
Conceptual illustration of the percentile-based slope-constraint mechanism applied to linear interpolation.

The output of Algorithm 1 is illustrated in Fig. 2, which provides an example of the slope-clipping process using the proposed constraint. In the real dataset, certain points generated by standard linear interpolation exhibit extreme values, raising concerns regarding potential overshooting. When these points are evaluated, if they meet the slope-constraint condition—meaning the slope at that point exceeds τ—the slope is clipped to the magnitude defined by our designated percentile. In this study, we utilized the 95th percentile slope, which is 15.70. Conversely, if the condition is false, no clipping occurs, and the original interpolated data point is retained for the imputation. In this study, the upper and lower slope boundaries are determined solely by the percentile, which serves as the only required parameter. Identifying the optimal percentile directly impacts the accuracy of the data imputation. Furthermore, the application of a percentile-based constraint allows the method to adapt to the unique characteristics of different datasets without requiring rigid parametric assumptions [27].

### Evaluation metrics and robustness analysis

The effectiveness of the proposed method was evaluated using R^2^, Mean Absolute Error (MAE) and Root Mean Square Error (RMSE). These are standard performance metrics widely utilized in data imputation and time-series forecasting research [7,22,23].

(9) $$R^{2}=1-\frac{\sum_{i=1}^{n}{\left(y_{i}-{\hat{y}}_{i}\right)}^{2}}{\sum_{i=1}^{n}{\left(y_{i}-{\bar{y}}_{i}\right)}^{2}}$$

(10) $$MAE=\frac{\sum_{i=1}^{N}\left|y_{i}-{\hat{y}}_{i}\right|}{N}$$

(11) $$RMSE=\sqrt{\frac{\sum_{i=1}^{N}{\left(y_{i}-{\hat{y}}_{i}\right)}^{2}}{N}}$$

R^2^ is used to measure the proportion of variance in the data that the proposed method can explain. In this context, $y_{i}$ represents the ground truth, ${\hat{y}}_{i}$ represents the imputed PM2.5 value, and ${\bar{y}}_{i}$ represents the average of the observed data. The performance is interpreted that an $R^{2}$ value approaching 1 indicates that the proposed imputation method effectively preserves the original data structure. Conversely, a value approaching 0 suggests a high level of error in the estimation. For MAE and RMSE, the interpretation follows a similar logic. Both metrics measure the deviation between the imputed values and the ground truth. For these indicators, a lower value signifies higher efficiency and accuracy in the data imputation process.

## Method validation

### Primary experiment

The primary objective of this experiment is to evaluate the performance of the proposed method in comparison to baseline techniques, including Linear Spline, Quadratic Spline, and Cubic Spline, across varying levels of data missingness. The results demonstrate that at low missingness levels (5–15%), the proposed method consistently yields the highest R^2^, while quadratic and cubic splines fail to provide a significant improvement over the standard linear approach (Table 1).

**Table 1:** Performance comparison of linear, quadratic, cubic, and proposed methods (Missing Rate 5%).

Method	R²	MAE	RMSE
Linear	0.831	3.76	6.00
Quadratic	0.842	3.65	5.82
Cubic	0.838	3.70	5.88
**Proposed**	**0.853**	**3.53**	**5.60**

**Fig. 3:**
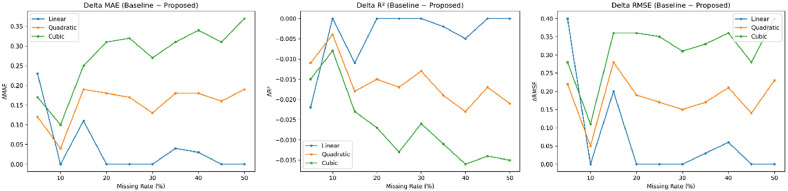
Performance differences between the proposed method and baseline interpolation methods across different missing rates.

**Fig. 4:**
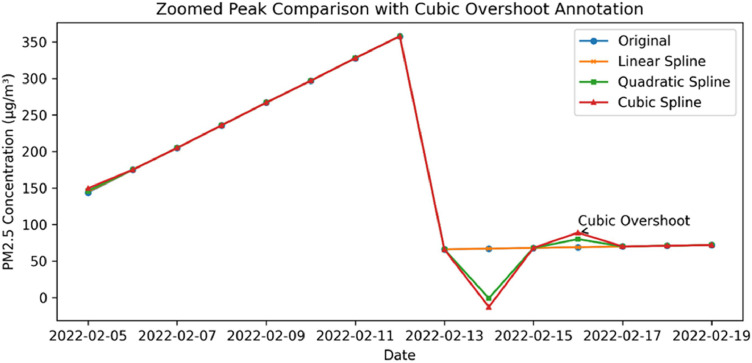
Example of cubic spline overshoot near a sharp PM2.5 peak compared with linear and quadratic interpolation.

For moderate missingness (20–35%), both quadratic and cubic splines exhibit a more rapid decline in performance compared to linear methods. This trend reflects the characteristic issues of overshooting and oscillation inherent in higher-order splines [1,28], as illustrated in Fig. 3, Fig. 4

In contrast, Slope-Constrained Linear Interpolation effectively mitigates these issues by directly controlling the first-order derivative. At high missingness levels (40–50%), the performance gap between the linear and proposed methods narrows, reflecting the inherent structural limitations of univariate interpolation [29]. Nevertheless, higher-order splines continue to show significantly lower performance. These findings are consistent with established numerical analysis theory, which suggests that higher-order splines may increase instability when data exhibits high discontinuity, particularly when missing values cluster into large gaps [2,29] (Tables 2 and 3).

**Table 2:** Sample imputation performance of the proposed method (5% Missing).

Date	Original	Imputed (Proposed)	Error	%Error
2024-01-05	143.0	143.33	0.33	0.23
2024-02-09	267.0	266.5	-0.5	-0.19
2024-02-10	297.0	297.0	0.0	0.0
2024-04-06	177.0	176.5	-0.5	-0.28
2024-04-08	238.0	197.0	-41.0	-17.23

**Table 3:** Performance of the proposed slope-constrained linear method under varying missing rates.

Missing (%)	R²	MAE	RMSE
5%	0.853	3.53	5.60
10%	0.865	3.19	4.90
15%	0.862	3.42	4.99
20%	0.720	4.07	6.44
25%	0.765	3.73	5.45
30%	0.822	3.25	4.52
35%	0.821	3.60	5.55
40%	0.764	4.16	5.89
45%	0.756	3.98	5.35
50%	0.553	4.43	6.75

### Parameter selection and sensitivity analysis

This experiment was conducted to investigate the percentile parameters at 80%, 85%, 90%, 95%, and 99% to evaluate how the level of constraint strictness affects data imputation performance. The Parameter Sensitivity Analysis was performed in conjunction with the Progressive Missing Rate Evaluation to determine the optimal parameter values across all missing rate scenarios. An example of these experimental results is presented in Table 4, which illustrates the Parameter Sensitivity Analysis specifically at a 20% missing rate.

**Table 4:** Sensitivity analysis of percentile-based slope threshold (Missing Rate 20%).

Percentile	R²	MAE	RMSE
80%	0.712	4.18	6.57
85%	0.716	4.12	6.50
90%	0.719	4.09	6.46
**95%**	**0.720**	**4.07**	**6.46**
99%	0.718	4.10	6.48

**Fig. 5:**
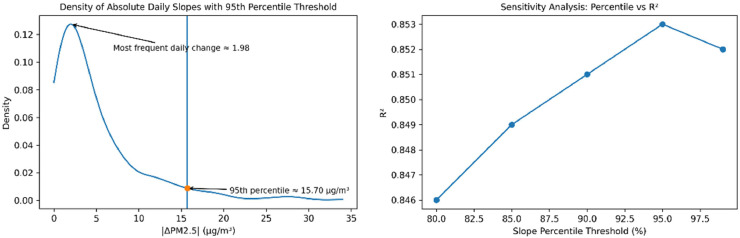
Empirical distribution of absolute daily PM2.5 slopes and the 95^th^ percentile threshold (left), and sensitivity of R² performance to percentile threshold selection (right).

**Fig. 6:**
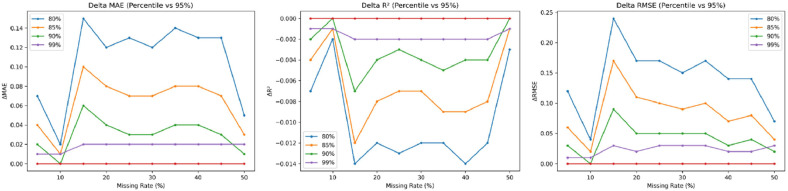
Performance deviation from the 95th percentile slope threshold across different missing rates.

The sensitivity analysis results indicate that at low missing rates (5–15%), increasing the percentile from 80% to 95% leads to a slight upward trend in R^2^, while MAE and RMSE consistently decrease. This reflects that an overly strict slope constraint (80%) may diminish the model's ability to track actual data trends, aligning with the bias–variance trade-off in model constraint management [21,30]; specifically, excessive constraints increase bias, whereas a more relaxed constraint (95%) provides a superior balance. For moderate missing rates (20–30%), the results across all percentiles are highly similar, with negligible R^2^ differences of less than 0.01. The increase in missing data at this level likely leads to missing data blocks, which is consistent with the findings of Junninen et al. [26] and Mariethoz et al. [30], who reported that the effectiveness of structural constraints diminishes as data gaps lengthen. Regarding performance at high missing rates (40–50%), the differences between percentiles become statistically insignificant, demonstrating that the method is stable under degradation (Figs. 5 and 6).

Based on this parameter sensitivity analysis, this research adopts the 95^th^ percentile as the standard value for the slope constraint. This setting consistently delivers peak or near-peak performance across all missing rate levels and exhibits robust stability against increasing data loss.

### Constraint only vs clipped extreme only

**Fig. 7:**
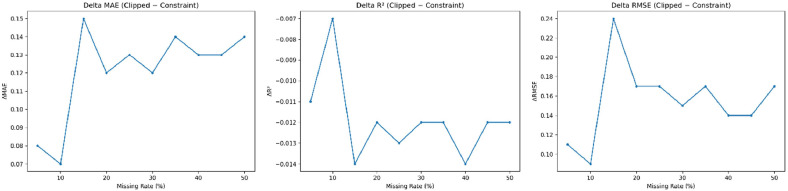
Ablation analysis comparing sequential slope constraint and global extreme-value clipping strategies.

For Constraint Only vs Clipped Extreme Only, the result shows that in the range of low missingness (5–15%), the Constraint Only slope limitation yields a higher R^2^ and consistently reduces MAE and RMSE compared to Clipped Extreme Only, suggesting that controlling slope limitation via slope distribution directly preserves temporal structures more accurately than simple value clipping, which fails to govern dynamic characteristics (Table 5 and Fig. 7).

**Table 5:** Ablation comparison between slope constraint and extreme-value clipping methods (missing rate 5%).

Method	R^2^	MAE	RMSE
Clipped Extreme Only	0.842	3.61	5.71
**Constraint Only**	**0.853**	**3.53**	**5.60**

This observation aligns with the principle that controlling the first-order derivative is essential for maintaining data features [31,32]. While performance differences remain minor during moderate missingness (20–35%), the Constraint Only method maintains its superior performance even at high missingness levels (40–50%), despite an overall decline in metrics; this indicates that slope-aware constraints act as an implicit regularization, consistent with robust time-series modeling theories where managing gradient structures reduces error amplification [31,33].

The experimental results demonstrate that the proposed Percentile-based Slope-Constrained Linear Interpolation consistently outperforms traditional linear interpolation for PM2.5 imputation. The effectiveness of the proposed method can be explained by the statistical characteristics of PM2.5 time-series data. In highly volatile environmental data, the distribution of daily slope changes typically exhibits a heavy-tailed behavior, where most slope values are relatively small while extreme changes occur infrequently. By constraining the interpolation slope using a high percentile threshold (e.g., the 95^th^ percentile), the proposed method effectively suppresses unrealistic gradient transitions that are unlikely to occur in real atmospheric processes. This mechanism acts as a structural regularization, preventing error amplification while still preserving the dominant temporal dynamics of the original time series, particularly when the missing data rate exceeds 15%. This confirms that the slope-limiting mechanism plays a critical role in preventing overshooting between data points, thereby significantly improving data fidelity. One of the most significant advantages of the proposed method is its computational efficiency. With a complexity of only O(n), the algorithm requires minimal resources, enabling real-time or near real-time processing. This makes it highly suitable for government agencies or environmental organizations that need to process data from multiple monitoring stations simultaneously. Furthermore, as a deterministic algorithm, it eliminates the need for model training and removes the risks of overfitting or concept drift, which are common challenges in machine learning-based approaches. From a theoretical perspective, controlling the slope distribution functions as a form of structural regularization. This aligns with established concepts in robust time-series modeling, which aim to limit the influence of outliers and reduce error amplification [34,33]. The integration of this method into existing data management pipelines is seamless and does not disrupt core operational structures. It can function effectively as either a pre-processing or post-processing step for data validation before the information is used for further analysis or released to the public.

## Limitations

Although the proposed method improves PM2.5 imputation accuracy compared with standard linear interpolation, several limitations remain.

First, this study used one year of data from a single monitoring station in Bangkok. While the dataset captures typical fluctuations in PM2.5 concentrations, including both high- and low-pollution periods, it does not represent long-term climatic variability. Large-scale climatic factors, such as El Niño–Southern Oscillation (ENSO) events or changes in regional emission patterns, may influence PM2.5 dynamics. Future work should extend the dataset to multiple years and additional monitoring stations to further evaluate the temporal robustness of the method.

Second, missing data were simulated using a progressive missing-rate scenario under the Missing Completely at Random (MCAR) assumption. In practice, monitoring stations often experience block missing patterns caused by sensor failure, maintenance downtime, or extreme weather conditions. Future studies should therefore investigate the performance of the proposed method under more realistic missing data patterns, including consecutive gaps.

Third, the proposed slope-constraint method relies on a single parameter derived from the percentile of observed value changes. Although this design keeps the method simple and computationally efficient, determining a universally optimal percentile threshold remains challenging. Future research may explore adaptive or dynamic percentile selection that adjusts to seasonal variability or changing data volatility.

Overall, extending the temporal scope and incorporating more realistic missing-data scenarios will help further validate the applicability of the proposed approach for practical air quality monitoring systems.

## Ethics statements

This research did not involve human participants, animal experiments, or social media data. The PM2.5 dataset used in this study was obtained from publicly available air quality monitoring records provided by the Thailand Pollution Control Department.

## CRediT authorship contribution statement

**Sawet Somnugpong:** Conceptualization, Methodology, Software, Investigation, Visualization, Supervision, Validation. **Narut Butploy:** Data curation, Formal analysis, Investigation, Writing – review & editing. **Kanokwan Khiewwan:** Data curation, Formal analysis, Investigation, Writing – original draft.

## Declaration of competing interest

The authors declare that they have no known competing financial interests or personal relationships that could have appeared to influence the work reported in this paper.

## Data Availability

Data will be made available on request.

## Appendix Supplementary materials

Image, application 1
